# Re-boot: Simulation Elective for Medical Students as Preparation Bootcamp for Obstetrics and Gynecology Residency

**DOI:** 10.7759/cureus.2811

**Published:** 2018-06-14

**Authors:** Veronica Lerner, Erin E Higgins, Abigal Winkel

**Affiliations:** 1 OBGYN, Montefiore Medical Center, Bronx, USA; 2 OBGYN, NYU Medical Center, New York, USA

**Keywords:** bootcamp, obgyn, medical students, simulation, prepare for residency

## Abstract

Objective: To evaluate the impact of a simulation-based elective on medical student preparedness for obstetrics and gynecology (OB/GYN) residency.

Methods: A two-week, simulation-based elective course for post-clerkship medical students was developed, and 10 students participated at a single academic institution in 2016 and 2017. Using standardized patients and team-based training, students practiced procedural and surgical skills, as well as the diagnosis, management, and work-up of commonly seen problems. Close coaching with a low student-faculty ratio was employed for each session, allowing for individualized feedback in real time. Prior to and after completing the elective, student knowledge was evaluated using the Preparation for Residency Knowledge Assessment tool (PrepForRes). Written course evaluations were also completed by students at the end of the course.

Results: Mean scores on the PrepForRes exam increased from 63.6% to 75.3% (p=0.0136). Notably, the average post-course score improved to a passing level, and all but one student achieved a passing score on the post-course test. Course evaluations and student feedback showed high satisfaction rates with the course.

Conclusions: This study demonstrates that a simulation-based elective course is an effective tool for helping medical students transition to OB/GYN residency. As medical schools work to facilitate the transition from undergraduate to graduate medical education, simulation can bridge gaps during this transition in order for students to meet entry-level residency requirements.

## Introduction

Initiation in the career of an obstetrics and gynecology (OB/GYN) resident represent a difficult period of learning and adjustment. Simulation has been used to aid with such transitions, as the journey from medical school to residency is fraught with challenges for doctors and risks for patient safety [1]. In order to better ensure that residents obtain the skills and knowledge necessary for effective care of patients, the Accreditation Council for Graduate Medical Education (ACGME) established educational milestones that describe specialty-specific benchmarks of competency [2-3]. It is expected that physicians entering training will have achieved Level One upon graduation from medical school [3-4].

Several medical schools and residency programs have instituted boot camps and residency preparation courses in an effort to ensure that new interns are ready for the challenges they will face in the learning environment [4-10].

In an effort to assess the medical knowledge of incoming interns in OB/GYN, the Association of Professors of Gynecology and Obstetrics (APGO) developed the Preparation for Residency Knowledge Assessment tool (PrepForRes) in 2014. PrepForRes is a written exam consisting of 107 multiple-choice questions created to assess medical knowledge based on ACGME Level One milestones [11]. However, there still does not exist a formal standardized curriculum preparing fourth-year medical students for the transition to OB/GYN residency, nor is there a subjective framework for accessing clinical performance [12]. The preparedness of OB/GYN residents for fellowship and independent practice as measured by their ability to function independently in various clinical settings has been recently addressed by a survey of accepting fellowship program directors, but no such data exists for graduating medical students or incoming interns to access their preparedness for residency [13].

In order to help prepare medical students for OB/GYN residency, simulation and clinical instructors at our institution developed a two-week, simulation-based elective course in 2016. This course was aimed at graduating students entering OB/GYN residencies, but it was also open to students preparing for fourth-year electives in OB/GYN and students pursuing other specialties who wanted to hone their skills and knowledge in women’s health. The curriculum consisted of multiple sessions based on specific learning objectives, including surgical skills, team-based training, and sessions utilizing standardized patients with extensive debriefings to practice the diagnosis, management, and work-up of commonly encountered problems.

The goal of the elective was to provide hands-on, simulation-based training to medical students to improve medical knowledge, procedural skills, teamwork, communication, and professionalism. We used the established PrepForRes exam as a proxy for medical knowledge to evaluate if this elective is effective at teaching entry-level knowledge expected of new OB/GYN interns. Additionally, students were surveyed after completion of the course to assess how this course affected their understanding of common topics, approach to learning, and overall preparedness for residency.

## Materials and methods

Third and fourth-year medical students who completed OB/GYN clerkship were invited to enroll in the two-week elective course in spring 2016 and 2017. Our inclusion criterion is third and fourth-year medical students who completed OB/GYN clerkship. We did not exclude any students. Six students enrolled in 2016 and four students enrolled in 2017, providing a sample of 10 students. Individual sessions with the corresponding teaching modality and student-faculty ratios as used in 2017 are listed in Table 1. Minor refinements to the curriculum used in 2016 based on faculty consensus and student feedback were implemented in 2017 course. We made the following changes: increased number of suturing and knot tying sessions and broke them up into shorter and more frequent time slots and added self-study and deliberate practice time to allow students to prepare and review material taught in sessions. In addition, in order to allow for above changes to take place, we removed EMR (electronic medical record) and note writing sessions, and made clinical shadowing opportunities optional. The Level One milestones taught in the course are listed in Table 2. All 10 students took PrepForRes both prior to and after finishing the elective course and completed written course evaluations at the end of the course.

**Table 1:** Curricular sessions by topic and teaching modality

Teaching Session	Teaching Modality	Time Spent (Hours)	Day of the elective	Student-to-Faculty Ratio
Suturing and knot tying, incision closure	Skills station	Multiple sequential sessions (self-study and coaching), followed by a formal assessment test. Seven hours of directed coaching, excluding self-study time.	1,3,5,8,10	2:1
Labor management (cervical exam, pelvimetry, AROM, FHR interpretation)	Didactic, skills station, simulated case with SP	2	2	3:1
Postpartum hemorrhage	Didactic, skills station, simulated case with SP	4	5	3:1
Difficult conversations, disclosure of errors	Narrative medicine	1	3	4:1
Preeclampsia	Didactic, skills station, simulated case with SP	4	6	3:1
Contraception placement (IUD, implant)	Didactic, skills station, simulated case with SP	4	4	3:1
Colposcopy/LEEP	Didactic, skills station	3	8	2:1
Ectopic pregnancy	Didactic, simulated case with SP	3	9	3:1
Principles of electrosurgery	Didactic, skills station	2		4:1
Cesarean section	Skills station	1	3	2:1
Circumcision	Skills station	1	7	2:1
2nd-degree laceration repair	Skills station	1	8	2:1
Ultrasound	Skills station	1	7	2:1
Hysteroscopy/cystoscopy	Didactic, skills station	3	9	2:1
Shoulder dystocia	Skills station, simulated case with SP	4	10	4:1

**Table 2:** ACGME Level One milestones taught in OB/GYN simulation elective ACGME: Accreditation Council for Graduate Medical Education; OB/GYN: obstetrics and gynecology.

Competency	Topic	Level 1 Milestone
Patient Care	Antepartum Care and Complications of Pregnancy	Demonstrates basic knowledge of normal obstetrical care and common medical complications seen in pregnancy.
	Care of Patients in the Intrapartum Period	Demonstrates basic knowledge of routine/uncomplicated intrapartum obstetrical care including, conduct of normal labor.
	Care of Patients in the Postpartum Period	Demonstrates basic knowledge of normal postpartum care.
	Obstetrical Technical Skills	Demonstrates basic surgical principles, including the use of universal precautions and aseptic technique. Performs basic procedures, including speculum examination and cervical examination.
	Gynecology Technical Skills: Laparotomy	Demonstrates knowledge of basic abdominal and pelvic anatomy. Demonstrates basic surgical principles, including the use of universal precautions and aseptic technique.
	Gynecology Technical Skills: Endoscopy	Demonstrates basic surgical principles, including the use of universal precautions and aseptic technique.
	Family Planning	Verbalizes basic knowledge about common contraceptive options.
	Ambulatory Gynecology	Demonstrates basic knowledge about common ambulatory gynecologic problems.
Medical Knowledge	Peri-Operative Care	Demonstrates knowledge of basic abdominal and pelvic anatomy.
	Health Care Maintenance and Disease Prevention	Demonstrates knowledge of the characteristics of a good screening test. Demonstrates knowledge of indications and limitations of commonly used screening tests.
Professionalism	Compassion, Integrity, and Respect for Others	Understands the importance of compassion, integrity, and respect for others. Demonstrates sensitivity and responsiveness to patients.
Interpersonal and Communication Skills	Communication with Patients and Families	Demonstrates adequate listening skills. Communicates effectively in routine clinical situations.

Additionally, PrepForRes was administered to all eight incoming first-year OB/GYN residents prior to the start of their intern year in 2017. Administration of this exam has become a standard process at our institution to characterize baseline knowledge and identify areas for professional growth.

## Results

When comparing pre-course and post-course exam scores for 10 students who participated in the 2016 and 2017 electives, mean test scores increased significantly from 63.45% to 75.20% (p=0.013) (Table 3). Notably, the average post-course score improved to a passing level (which is defined as 70% or above) from an average pre-course score that was in the failing range. All but one student achieved a passing score on the post-course test, which corresponds with a 90% pass rate overall.

**Table 3:** Pre-course and post-course exam scores for students completing OB/GYN simulation elective in 2016 and 2017 PrepForRes: Preparation for Residency Knowledge Assessment tool; OB/GYN: obstetrics and gynecology.

Student Number	Pre-Course PrepForRes Exam Score (%)	Post-Course PrepForRes Exam Score (%)
1	35	59
2	65	73
3	70	82
4	70	80
5	73	78
6	72	77
7	51	71
8	64	77
9	67	79
10	69	77
Mean Score	64	75 (p = 0.0136)

Course evaluations, individual session evaluations, and student feedback also showed high satisfaction with the course. The course was scored highly with regards to usefulness in preparation for residency. For example, one participant noted that “this course made me feel so much more comfortable and ready for residency,” while another stated that “these two weeks were the most valuable and enjoyable two weeks I've had in medical school.” Students reported improvement in their communication skills, both with patients and families and other members of the healthcare team. Students also valued the use of simulation and hands-on experience: “I was able to see how much of a difference simulation and utilization of the skills lab makes.” Finally, a majority (75%) of students felt that this course should be mandatory prior to starting residency, and all indicated that they would take the course regardless of obtaining credits to apply towards graduation.

Scores from the incoming first-year cohort of OB/GYN interns in 2017 were compared to the post-course exam scores of students who completed the elective (Table 4). One first-year resident had participated in the elective previously and was excluded from analysis. The mean test score of the remaining seven interns was 76 %, which was similar to the post-elective mean test score (p=0.7701). Notably, two subjects in the resident cohort did not achieve passing scores, which represents a 75% pass rate overall. 

**Table 4:** Exam scores for incoming first-year residents in 2017 PrepForRes: Preparation for Residency Knowledge Assessment tool.

Intern Number	PrepForRes Exam score (%)
1	70
2	81
3	75
4	75
5	74
6	69
7	90
Mean Score	76

## Discussion

This pilot study demonstrates that an intensive, simulation-based elective is well received by medical students and is effective at teaching the basic medical knowledge expected of incoming OB/GYN residents.

Our study does have several limitations. First, this pilot study is limited by a small sample size of 10 students at a single institution. Moreover, taking the same exam twice in a row, two weeks apart, might be problematic because an improvement in scores might be due to students memorizing correct exam answers and studying to understand why wrong answers were chosen, not directly related to learning from elective activities. However, there was no validated alternative exam that we could use to address this issue. Additionally, instructors were known to the students, which may influence course evaluations. It is further limited by the confines of PrepForRes to test only medical knowledge as measured by a multiple-choice exam, in contrast with performance in a simulated or a clinical setting [14]. It is also important to note that significant resources were dedicated to this elective, and cost considerations need to be addressed in future studies.

With regard to the PrepForRes scores of incoming interns compared to those of medical students, it is important to consider that selection bias may have been responsible for the absence of statistical significance. More specifically, incoming interns in our program are selected based on several criteria, one of which is high scores on the United States Medical Licensing Exam (USMLE). Good performance on the USLME is likely to correlate with better performance on multiple-choice exams, such as PrepForRes. Our residency program is also highly competitive, exacerbating this effect on selection bias, as incoming interns might be more motivated to learn and are better performers than graduating medical students. It is possible that if students who took the elective and interns who entered our program were “adjusted” for their ability to do well on the multiple-choice exam and their competitiveness, the actual effect of the elective on their medical knowledge would have been more profound.

Future work remains to be done to understand how this elective could impact clinical performance and patient outcomes. Currently, there is no standardized, proficiency-based assessment for use during residency training to measure competency, similar to the system utilized in residency training in England, and direct observations such as entrustable professional activities (EPAs) and objective structured assessment of technical skills (OSATs) are not widely implemented [15]. Having a more robust, proficiency-based framework for the evaluation of clinical performance might provide better metrics for the evaluation of interventions such as our elective, rather than having to rely on proxies such as PrepForRes.

Administration of PrepForRes at various time intervals (e.g., three and six months after completion of the course) could assess retention of knowledge over a longer period of time. As the deterioration of skill over time is of concern, the timing of this course needs to be explored in future studies. It may be more beneficial to enroll incoming interns in this boot camp, as has been done in orthopedic residency programs. Expansion to multiple study sites would also improve the generalizability of these results.

## Conclusions

A great need exists to adequately prepare students for residency. While many medical schools and residency programs have instituted generic and specialty-specific immersion courses and boot camps, there is much variation with regards to the teaching modality, course content, and length of programs. As demonstrated by this study, simulation represents an exciting avenue to both teach and evaluate residency preparedness in a standardized manner and could be employed across a wide variety of disciplines.
